# Paradoxical Agitation and Masseter Spasm During Propofol Procedural Sedation: A Case Report

**DOI:** 10.5811/cpcem.21283

**Published:** 2024-11-02

**Authors:** Amit S Padaki, Prabhdeep Uppal, Michael Perza

**Affiliations:** * Baylor College of Medicine, Department of Emergency Medicine, Houston, Texas; † Christiana Care Health System, Department of Emergency Medicine, Newark, Delaware

**Keywords:** propofol, adverse event, agitation, excitotoxicity, opisthotonos, case report

## Abstract

**Introduction:**

Propofol is an anesthetic agent commonly used in emergency department (ED) procedural sedation. It is often preferred in orthopedic procedures because of its muscle-relaxing properties. Rarely, however, it can induce agitation and muscle hypertonicity.

**Case Report:**

A 58-year-old man presented to the ED with a left ankle fracture-dislocation. Propofol was used to facilitate procedural sedation, but the patient became mildly agitated. Ketamine was used to achieve full induction, after which propofol was used again to facilitate muscle relaxation. Near the end of the procedure, the patient had opisthotonos and masseter spasm requiring bag-valve-mask ventilation and subsequent intubation. This reaction was ultimately attributed to adverse effects of the propofol.

**Conclusion:**

While propofol is generally well tolerated, it can potentially cause agitation, hypertonicity, and other side effects such as muscle spasms and seizure-like activity. Acknowledging and preparing for these risks can potentially improve patient outcomes.

## INTRODUCTION

Propofol sedation is commonly used in the emergency department (ED) for multiple indications, including fracture reduction and post-intubation sedation. Propofol’s predominant mechanism of action appears to be through gamma-aminobutyric acid (GABA) receptors in the central nervous system, although some additional effects may include inhibition of N-methyl-D-aspartate receptors^1^ and modulation of slow calcium channels.^2^ Propofol has both quick onset and offset, allowing it to be used for procedures of relatively short duration. It has the added benefit of muscle relaxation and is further used for its antiepileptic and neuroprotective properties. Propofol’s side effects include bradycardia, hypotension, and respiratory depression. While potentially serious, the risk of these adverse effects can be mitigated through thoughtful dose titration and appropriate monitoring.^3^ Unfortunately, propofol may also cause agitation and muscle hypertonicity, directly opposite to its intended effects. We describe such a case below.

## CASE REPORT

A 58-year-old man presented to the ED with a left ankle fracture-dislocation. The patient had tripped over his walker the prior evening. He then visited a medical aid unit the next morning, where radiographs revealed a comminuted and displaced distal fibular fracture with tibiotalar dislocation. He was transferred to the ED for additional evaluation.

In the ED, the patient had an obvious left ankle deformity with skin tenting to the medial malleolus and overlying necrosis. His foot was neurovascularly intact with a 2+ dorsalis pedis pulse, appropriate capillary refill, and the ability to flex, extend, and sense all his toes. He had no other injuries on exam. Vital signs were stable. The patient had a history of obesity and gait instability but otherwise no comorbidities. He took no medications and denied any history of drug or alcohol use. Due to skin tenting and overlying necrosis, the team elected to perform an emergent reduction. The patient had a Mallampati Score of 3, American Society of Anesthesiologists score of 2, and weighed approximately 140 kilograms. He denied any history of complications from anesthesia.

The sedation was initiated with 50 milligrams (mg) of intravenous (IV) propofol. The patient became agitated and disoriented, picking at the air. An additional 50 mg of IV propofol was administered, but he then began sitting up and moving. Because the second dose of propofol appeared to paradoxically increase the patient’s agitation, the team chose to change to ketamine. The patient received a trial dose of 30 mg of IV ketamine and tolerated this well. He then received an additional 100 mg of IV ketamine. The patient was then fully induced but continued to have significant muscle spasms preventing reduction.

Given his muscle spasms, the team elected to return to propofol for its muscle-relaxing effects. The patient received smaller doses of IV propofol, ultimately adding to a full induction dose of 130 mg. He remained hemodynamically stable throughout this procedure. His ankle was able to be reduced but remained somewhat loose and would subluxate in and out of position. The decision was made to place a splint and to defer further reduction to the orthopedics service.

Five to six minutes after the final propofol dose, while holding the splint, the patient was noted to have whole body flushing, warmth, and muscle spasms, including clenching of the jaw and arching of the back. His oxygen saturation dropped to the low 70s. Jaw thrust was applied, bag-valve-mask ventilation was initiated, and oxygen saturation improved to the high 80s. Despite 10 minutes of bagging, the patient did not have further improvement in mental status or oxygen saturation, and the decision was made to intubate. He was paralyzed with 150 mg of IV succinylcholine and intubated during the first attempt. After intubation, the patient’s oxygen saturation improved to 99%.

The orthopedist arrived at the bedside in the ED after intubation and reduced and re-splinted the patient’s ankle, which continued to re-dislocate. About 20 minutes after intubation, the patient’s muscle tension and spasms began to improve. He was admitted to the surgical intensive care unit and had an external fixation of the left ankle the following morning. The patient failed extubation on hospital day (HD) 1 but was subsequently extubated on HD 2 and discharged to a subacute rehab facility.

## DISCUSSION

We believe our patient exhibited signs of propofol excitotoxicity, specifically opisthotonos, a tetanic condition in which the spine hyperextends into a backward-arched position. A review of the literature shows several similar case reports of propofol causing myoclonus,^4^ opisthotonos,^5^ neuroexcitation,^6^ and even seizure-like activity.^7^

**CPC-EM Capsule**

What do we already know about this clinical entity?

*Propofol is an anesthetic agent used in procedural sedation; common side effects include hypotension and bradycardia.*

What makes this presentation of disease reportable?

*We report the unusual occurrence of agitation and masseter spasm in a patient who was administered propofol for an ankle fracture-dislocation.*

What is the major learning point?

*The mechanism underlying propofol causing agitation and muscle spasm is poorly understood. There is no specific treatment apart from supportive care.*

How might this improve emergency medicine practice?

*Awareness of these possible side effects can allow physicians to better recognize and respond to them.*

The precise pathophysiology of propofol’s neuroexcitatory properties remains unclear. One possibility is that propofol’s inhibition of glycine, an inhibitory neurotransmitter, may lead to excitatory pathway activation and diffuse muscle contractions. A second possibility is that falling propofol concentrations near the end of procedures may lead to decreased GABA activation and rebound excitation. This is supported by opisthotonos often occurring near the end of procedures or even after their conclusion, as was witnessed in our case. Literature on risk factors is limited. Prior studies do suggest propofol neuroexcitation may be more common in female patients^8^ and those with epilepsy.^7^ Emergence agitation following any type of anesthesia may be more common in obese patients and those with histories of alcohol and substance use disorders.^9^

There is currently no standard treatment regimen for propofol excitotoxicity. Some physicians have noted resolution after cessation or reduction of the propofol administration.^10^ Others have attempted symptomatic treatment with benzodiazepines^11^ or with anticholinergics such as benztropine.^12^ Of note, while most cases are self-limited and can be treated conservatively, some cases of excitotoxicity have been noted to be refractory to benzodiazepines and other rescue medications.^13^ If prolonged, such excitation may require intubation for airway protection, as was seen in our case.^14^

We should note that similar excitotoxic effects have been seen with other anesthetic agents, including ketamine.^15^,^16^ It appears that most ketamine reactions appear to occur quickly after administration, as the ketamine peaks. The reaction in our case occurred significantly after the ketamine was administered, making ketamine excitation less likely. The combination of propofol and ketamine may also lead to increased emergence agitation, although not necessarily hypertonia.^17^

## CONCLUSION

Propofol’s pharmacokinetics and risk profile have led to its increasing use in the ED setting. It is now used for procedural sedation and seizure management, as well as for indications as wide-ranging as alcohol withdrawal^18^ and migraine.^19^ Clinicians are classically taught that the risks of propofol include hypotension and bradycardia. Here, we describe a case of excitotoxicity, a nearly opposite adverse event. Awareness of this unusual phenomenon, including that it can occur even after procedures are concluded, could potentially improve patient safety and outcomes.
